# Validation of an instrument for perceived factors affecting fruit and vegetable intake based on Pender's health promotion model

**DOI:** 10.1017/jns.2021.90

**Published:** 2022-02-09

**Authors:** Freshteh Khatti-Dizabadi, Jamshid Yazdani-Charati, Reza Amani, Firoozeh Mostafavi

**Affiliations:** 1 Department of Health Education and Promotion, Faculty of Health, Isfahan University of Medical Sciences, Isfahan, Iran; 2 Department of Biostatistics, Centre Addiction Research Institutes, Mazandaran University of Medical Sciences, Sari, Iran; 3 Department of Clinical Nutrition, School of Nutrition and Food Sciences, Food Security Research Center, Isfahan University of Medical Sciences, Isfahan, Iran; 4 Department of Health Education and Promotion, School of Health, Isfahan University of Medical Sciences, Isfahan, Iran

**Keywords:** Factor analysis, Fruit and vegetable, Questionnaire, Validation, Validity, CVI, content validity index, CVR, content validity ratio, HPM, health promotion model, KMO, Kaiser-Meyer-Olkin, PCA, principal component analysis

## Abstract

Increasing fruit and vegetable (F&V) intake has a protective role against chronic conditions such as cardiovascular disease, cancer and diabetes. The present study aimed to validate an instrument for measuring the perception of effective factors on consumption behaviour of F&V based on Pender's health promotion model (HPM).This cross-sectional validation survey has consisted of five steps: literature review in order to plan and develop an instrument, face validity assessment, content validity assessment, reliability assessment and construct validity assessment with the cooperation of experts in health education, nutritionists and the target group (government employees). In the present study, reliability and validity of constructs were determined through Cronbach's alpha and exploratory factor analysis, respectively, in SPSS 22. The mean impact score was acceptable for 96·42 % of items in face validity. The mean scores of content validity ratio (CVR), content validity index (CVI) and reliability were 0·92, 0·97 and 0·96, respectively. According to the principal component analysis with varimax rotation, 104 items were identified in 15 factors contributing to 61·17 % of the model cumulative variance. Given the favourable scores of the research instrument in face validity, content validity and reliability as well as its ability to predict the extracted factors from the model, it can be used as a suitable instrument in future studies.

## Introduction

Healthy nutrition habits play an important role in preventing chronic diseases in later stages of life^(1)^. Various studies have reported the protective roles via increasing intake of fruits and vegetables (F&V: Fruits such as apples, oranges, bananas, watermelons, melons, grapes and pomegranates and Vegetables such as raw vegetables, cooked vegetables, potatoes, tomatoes, cucumbers and carrots) against chronic conditions such as cardiovascular disease, cancer and diabetes^(2–6)^. Low F&V intake is globally associated with 2·8 % of deaths, a considerable number of which are caused by cardiovascular disease, stroke and gastrointestinal cancer^(7)^. Therefore, increasing F&V intake is a part of the public health strategies of the World Health Organization (WHO) for preventing non-communicable diseases^(8)^. According to a study by Msambickaka, despite the benefits of F&V intake, more than 75 % of the world's population fail to consume enough F&V^(9)^. Based on the studies conducted in Iran, Zamanian pointed out that F&V intake was lower than the recommended amount (minimum of 400 g or 5 servings of F&V per day^(10)^)^(11)^. In a review study of Abdi *et al.* in the field of food basket and food consumption pattern of Iranians from 2000 to 2014, the results have showed that the average per capita consumption of F&V in Iran 142 and 286 g/d, respectively^(12)^, as well in Mazandaran province of Iran, according to the latest study, 70 and 49 % of people did not consume enough F&V (minimum of 2 servings F and 3 servings V), respectively^(13)^.

Benefits of consuming F&V in people's health based on epidemiological results for daily consumption are recommended^(14)^. For example, the results of a systematic review and meta-analysis of the dose-response of F&V to the risk of cardiovascular disease, cancer and mortality have indicated that 800 g/d consumption has reduced all cases, except cancer which has been reduced by consuming 600 g/d^(15)^.

Knowledge plays a key role in changing health behaviour; therefore, it seems necessary to publicise F&V intake required to maintain ideal health^(9)^. Pengpid *et al.* showed that targeted interventions could increase F&V intake by identifying changeable risk factors such as low levels of education and general obesity^(16)^. According to Tassitano *et al.*, self-efficacy in behaviour change strategies was one of the main predictors of F&V intake in Brazil^(17)^. In addition, Wilsher *et al.* introduced conscience and emotional stability as very strong dimensions of F&V intake in the UK with respect to the role and importance of personality in diet and health behaviour^(18)^. Thus, studies conducted in recent decades have focused mainly on perception of generation and maintenance of healthy behaviour^(17)^. The identification of mediation factors has an effective role in formulating intervention programmes^(19)^. One of such factors is to attract social support for healthy nutrition, which can be attributed to the positive effect and encouragement of others to have healthy eating habits^(20)^. Considering the fact that F&V intake can be correlated with economic and social status as well as cultural patterns in an environment^(9)^, appropriate cultural intervention is needed to target existing barriers to healthy diet and awareness of nutritional guidelines^(20)^. Psychologists have developed specific models and theories to explain the determinants of people's behaviours in order to understand their behaviours and choices of people in the aspects of everyday life such as nutrition; therefore, theories and models can help program developers to identify the most effective health-promoting behavioural determinants in a particular population^(21)^. For instance, Pender's health promotion model (HPM) has developed to predict health behaviour and included three groups of the effective factors health-promoting behaviour: individual experiences and characteristics, specific behavioural emotions and cognitions, and behavioural outcomes^(22)^. Although individual characteristics cannot be changed, other specific variables such as Behavioural perceptions and emotions can be changed through appropriate intervention, which results will be observed in Behavioural outcome including: commitment to planning and prioritisation of priorities and demands. In other words, Behavioural outcome will be the result of people's participation in health behaviour^(23)^. Also, considering that Pender's HPM has an ecological approach to change behaviour and takes into account interpersonal, interpersonal, organisational and social factors, it can be helpful in identifying the effective factors in creating and maintaining this behaviour^(24)^. Therefore, as a framework for planning health interventions, this model can be applied to improve health-promoting behaviour^(22)^. Adults not only consume less fruit and fewer vegetables than recommended^(11,25)^ but are also exposed to at least three out of five risk factors for chronic diseases, e.g. cardiovascular disease^(26)^; hence, it is necessary to design and validate a model-based appropriate instrument that would be able to make strong predictions about the factors affecting F&V intake behaviour. Given the protective effect of F&V intake against a variety of medical diseases, e.g. cardiovascular disease, it has been considered a public health priority in many countries^(27)^. Therefore, the present study aimed to validate an instrument to measure the perception of effective factors on F&V intake based on Pender's HPM among in a group of nutritionists, health education and promotion and the public sector employees, the offices of Ghaemshahr city of Mazandaran province in Iran were conducted.

## Methodology

### Subjects

**Fig. 1.**
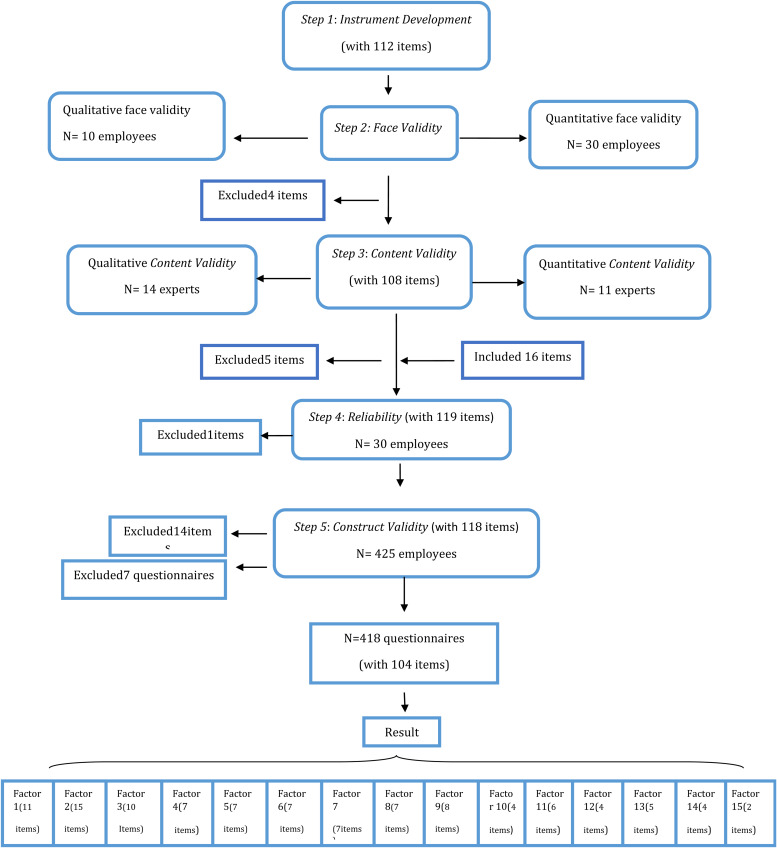
Flowchart of steps of the study.

In this cross-sectional study, 11 health education and health promotion specialists in the Step of Content Validity and 495 employees of the public sector in different Steps of the study (qualitative face validity: *n* 10, quantitative face validity: *n* 30, reliability: *n* 30 and Construct Validity: *n* 425) participated (Fig. 1).

### Steps of the study

The research spanned from 23 October 2018 to 23 July 2019 and in six steps: instrument development through literature review, face validity assessment, content validity assessment, reliability assessment, construct validity assessment and external validity.

#### Step 1 (instrument development)

In order to compile the instrument items, an extensive literature review was performed on F&V intake in Iran and abroad. Based on the appropriate items used in different instruments, a pool of questions was then prepared^(28–36)^. With the help of several health education and promotion experts, the new instrument items were finally designed in accordance with the constructs of Pender's HPM including the following factors: previous relevant behaviour, behaviour-related emotions, perceived barriers, perceived benefits, perceived self-efficacy, interpersonal influences, situational influences, commitment to action plan, immediate preferences and demand, motivational factors, and behavioural outcome. The items were then assessed on the four- and five-point Likert and Yes/No scales.

#### Step 2 (face validity)

Face validity was determined through qualitative and quantitative methods. First, ten people from the target group (government employees) were interviewed and asked to comment on the presence of difficult phrases, lexical ambiguity or different perceptions in each item of every construct and to express the appropriateness and coordination of items with the main purpose of each construct^(37)^. After the views of the target group were collected, necessary changes were made in the items. To check face validity, the instrument was then provided again for thirty members of the target group, who were then asked to rate each item in terms of importance on the Likert scale (*it does not matter at all; it does not matter; it almost matters; it matters* and *it matters very much*) from 0 to 5. The impact scores for the importance of points 5 and 4 were then calculated separately through ‘importance × relative frequency [%] = the item impact score’. Finally, the mean impact score of each item was determined, and items with an impact score of lower than the cut-off point of 1·5 were removed from the instrument^(38)^.

#### Step 3 (content validity)

Validity analysis depends on the test content logical analysis which is examined in terms of compliance with scientific literature, the use of appropriate words, writing principles and correct position of each item. In other words, it answers the question whether the instrument content can measure the defined purpose^(39)^. Therefore, if there is an agreement between individuals on validity, the instrument has content validity. In the present study, content validity was assessed through qualitative and quantitative methods. In the qualitative method, the instrument was sent to eighteen health education and promotion and nutrition experts for a careful review. Fourteen experts returned their written views. The quantitative method was employed to ensure the selection of the most important and correct content for the items regarding measurement of the intended objectives. Therefore, the content validity ratio (CVR) and the content validity index (CVI) were used. For this purpose, each research item was evaluated by eleven health education and promotion experts who had already announced their cooperation, based on a three-point Likert scale (1: necessary, 2: not necessary but useful and 3: not necessary). The CVR numerical value for each item was then determined through the following formula and the Lawshe table (Table 1).
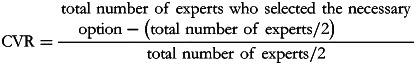

**Table 1.** The minimum acceptable content validity ratio (CVR) based on the number of participating experts to determine validity

Number of experts	Acceptable CVR	Number of experts	Acceptable CVR
5	0·99	13	0·54
6	0·99	14	0·51
7	0·99	15	0·49
8	0·75	20	0·42
9	0·78	25	0·37
10	0·62	30	0·33
11	0·59	35	0·31
12	0·56	40	0·29

According to the number of experts and the Lawshe table, a CVR of ≥0·59 was approved. To determine the CVI, the experts evaluated each item in terms of three criteria, i.e. simplicity, relevance and clarity, on a four-point Likert scale (1: quite simple, 2: simple, 3: relatively simple, 4: not simple; 1: fully relevant, 2: relevant, 3: relatively relevant, 4: irrelevant;1: fully clear, 2: clear, 3: relatively clear, 4: unclear). The CVI was then calculated for each item considering the total number of selected options of *completely agree* and *agree with each of the three criteria* (i.e. completely relevant and relevant) according to the following formula:
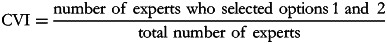


In this study, a CVI score of >0·79 was considered appropriate, whereas scores between 0·70 and 0·79 were questionable. Hence, each relevant item was reviewed and corrected according to the opinions and suggestions of experts, and scores <0·70 were not accepted, while the relevant items were removed from the instrument^(40,41)^. After the problematic items were reviewed, corrected and deleted, the modified instrument was provided for the experts, and their CVR and CVI were recalculated.

#### Step 4 (reliability)

Regarding internal stability or reliability, most instruments have different scales and dimensions; therefore, it must be ensured that the subscale items are homogeneous and measure similar properties^(42)^. Therefore, the instrument was provided for thirty members of the study target group (government employees). After each item was answered on four- and five-point Likert and Yes/No scales, the internal consistency of each construct of Pender's HPM was measured through Cronbach's alpha in SPSS 22. Since the reliability of at least 70 % is desirable in scientific references, a minimum score of 70 % was also accepted as the instrument reliability^(42)^.

#### Step 5 (construct validity)

Factor analysis is an important method for evaluating construct validity to determine the predictability of variables^(37)^; therefore, the sample size in this method should include three to fifty people per item according to Knapp and Brown^(43)^. Since a total of 118 items existed in the original research instrument, 3 people were selected for each item. Given a possible attrition rate of 20 %, 425 people were selected as the sample size. The subjects were selected through the random cluster sampling method. For this purpose, fifteen offices (about one-third of the offices) were randomly selected as clusters from the Governmental offices of Ghaemshahr, Mazandaran Province, Iran. These offices included Environment, Telecommunications, Technical and Vocational, Governor's office, Electricity, Red Crescent, Foundation, Agricultural Jihad, Civil and Personal status Registration, Document Registration, Labor and Cooperation, Sports and Youth, Roads and Urban Development, Industry and Mining and Social Security. The selected offices were located in different areas of the city with a variety of jobs and in turn differences in the amount of payment as a monthly salary. The individuals were then selected through the simple random sampling method from the designated offices. Inclusion criteria were employment in the designated offices and willingness to participate in the study after being reassured of information confidentiality. The exclusion criterion was reluctant to continue cooperation in completing the questionnaire or incomplete completion of questionnaire. There was no compulsion on participating in the study by the researcher or the heads of the offices. The participants responded to the perceived factors affecting the F&V intake behaviour questionnaire based on Pender's HPM with eleven constructs along with items of the knowledge section and socio-demographic characteristics in the self-report manner and due to the high number of instrument items, were collected after a few days (3–5 d).

##### Research instrument properties

The questionnaire consisted of the following components:

1. *Socio-demographic characteristics* included items about age, gender, education, etc.
2. *Knowledge section* aimed to determine the knowledge of individuals about the daily amounts and health benefits of F&V intake by choosing the correct option with a score of one. Based on the average number of correct knowledge answers, individuals were assessed with six items in score range 0–6.
3. *Previous relevant behaviour* intended to examine the previous relevant F&V intake behaviour and habits as well as their frequency during at least the past 1 month. This component had eight items designed on a four-point Likert scale (0: never and 3: always) in score range 0–24.
4. *Perceived self-efficacy* pertained to the competence of individuals to organise and perform the F&V intake behaviour as well as self-confidence in successfully performing this health behaviour. This component had eleven items designed on a five-point Likert scale (0: strongly disagree and 4: strongly agree) in score range 0–44.
5. *Behaviour-related emotions* pertained to abstract feelings and emotions that occurred before, during and after the F&V intake behaviour. This component had ten items designed on a five-point Likert scale (for items with a positive response, 0: never and 4: very much; and for items with a reverse negative response, 4: never and 0: very high) in score range 0–40.
6. *Perceived benefits* pertained to the perception of positive or reinforcing results of the F&V intake behaviour. This component had seven items designed on a five-point Likert scale (0: strongly disagree and 4: strongly agree) in score range 0–28.
7. *Perceived barriers* pertained to the perception of barriers, difficulties and personal costs regarding the F&V intake behaviour. This component had seventeen items designed on a five-point Likert scale (4: never and 0: very much) in score range 0–68.
8. *Interpersonal influences* included expectations or encouragement of other people about the F&V intake behaviour, designed in two separate sections. The first section pertained to the statements about expectations or encouragement of different people about the F&V intake behaviour, while the second section concerned the importance of people's opinion regarding the decisions made to perform the F&V intake behaviour. This component had seven items in each section designed on a five-point Likert scale (0: not at all and 4: always) in score range 0–56.
9. *Situational influences* pertained to perceptions about the ability of the living environment (situations, places and social events) to perform the F&V intake behaviour, designed in three separate sections. The first section consisted of four items, designed on a five-point Likert scale (0: never and 4: very much) about the effects of situations such as training classes or media on the desire to consume fruits and vegetables. Each of the second and third sections had seven items, designed on a five-point Likert scale (0: never and 4: very much) about the effects of different places and social events on the F&V intake behaviour in score range 0–72.
10. *Motivational factors* referred to factors (external and internal stimuli) that motivated people to perform F&V intake behaviour. This component had nine items, designed on a five-point Likert scale (0: not important at all and 4: very important) in score range 0–36.
11. *Commitment to the action plan* pertained to commitment to the F&V intake behaviour; this component had four items (two for fruit intake and two for vegetable intake), designed on the Yes/No and the three-point Likert scale (0: not at all and 2: very much) in score range 0–6.
12. *Immediate preferences and demand* included food preferences in case of simultaneous access to fruits, vegetables and other foods. This component had eight items, designed on the Yes/No scale in score range 0–8.
13. *Behavioural outcome* referred to the final desired behaviour or consequence of the decision and readiness for action on F&V intake. This component had six items, designed on a 5-point Likert scale (0: not at all and 4: always) in score range 0–24. The score range in the instrument was 0–412.

To confirm the construct validity, the variables with an internal correlation were grouped through exploratory factor analysis. For this purpose, the two indicators of Kaiser-Meyer-Olkin (KMO) and Bartlett's test of sphericity were employed to evaluate data appropriateness for factor analysis. KMO is a test that checks the sample size adequacy. It varies between zero and one. The closer the resultant number to one, the more probable the sample size adequacy for factor classification is to be confirmed. The Bartlett's test of sphericity determines whether the obtained correlation matrix is significantly different from zero; in other words, it analyses the existence of correlation between the instrument items for their integration^(44,45)^. After the correlation matrix of variables was created, the factors were extracted through principal component analysis (PCA) with varimax rotation. Finally, the highly correlated variables were placed in a group or factor. For better results, a cut-off point of 0·40 was used for high correlation between research variables. The exploratory factor analysis was performed in SPSS 22.

#### Step 6 (external validity)

External validity is used to examine whether the results obtained from the internal validity of the instrument can be generalised to a similar group to the study group or a larger group. Because an instrument may have good internal credibility but not be generalisable to larger groups or communities^(46)^. Therefore, in the present study, the demographic characteristics of the target group with the general population characteristics of Iran (Table 3), which were extracted from the results of the latest population and housing census in the country^(47)^ based on One-Sample *T*-Test, One-Sample Binomial Test and One-Sample Chi-Square Test were compared and analyzed.

### Ethics

This study was approved by Isfahan University of Medical Sciences with number 398521and ethics code IR.MUI.RESEARCH.REC.1398.465. All participants in this study completed and signed the consent form before participating in the various steps of the study.

## Results

### Step 1 (instrument development)

A total of 112 items were designed for the primary instrument. Out of 112 items, 8 pertained to the knowledge section, 9 to the construct of previous relevant behaviour, 11 to the construct of perceived self-efficacy, 9 to the construct of behaviour-related emotions, 9 to the construct of perceived benefits, 14 to the construct of perceived barriers, 14 to the construct of interpersonal influences, 11 to the construct of situational influences, 9 to the construct of motivational factors, 4 to the construct of commitment to action plan, 8 to the construct of immediate preferences and demand and 6 to the construct of behavioural outcome.

### Step 2 (face validity)

According to the face validity test results, after the results of the interview with the target group were applied to correct and revise some of the items in the qualitative section of face validity (in terms of ambiguity, simplification and higher coordination of the items), an impact score of <1·5 cut-off point was obtained by one item in the quantitative part of face validity, one item in the knowledge section, and one and two items in the constructs of previous relevant behaviour and perceived benefits, respectively; 96·42 % of the items had an acceptable average impact score and 3·57 % of items dropout of the questionnaire. The highest mean impact score pertained to three items in construct of motivational factors with a total score of 2·5; the items were (1) ‘How important is healthiness to motivate you more to eat fruits and vegetables?’ (2) ‘How important is freshness to motivate you more to eat fruits and vegetables?’ (3) ‘How important is proper price to motivate you more to eat fruits and vegetables?’ The lowest mean impact score (0·40) pertained to one item in the construct of perceived benefits, which was ‘If I eat fruits and vegetables every day, I will be accepted by others’ (Table 2).

**Table 2.** The mean content validity ratio (CVR), content validity index (CVI), impact score and Cronbach's alpha coefficient of Pender's HPM constructs

Row	Pender's HPM constructs	CVI	CVR	Cronbach's alpha coefficient	Impact score
1	Knowledge	0·97	0·89	0·73	1·98
2	Previous relevant behaviour	0·93	0·90	0·85	1·77
3	Perceived self-efficiency	0·93	0·85	0·90	1·77
4	Behaviour-related emotions	0·90	0·89	0·92	1·63
5	Perceived benefits	0·98	0·94	0·89	1·78
6	Perceived barriers	0·98	0·92	0·92	1·72
7	Interpersonal influences	0·99	0·98	0·85	1·62
8	Situational influences	0·99	0·94	0·88	1·68
9	Incentive factors	1·00	0·95	0·71	2·12
10	Commitment to the action plan	1·00	1·00	0·90	1·78
11	Immediate preferences and demand	0·97	0·85	0·74	1·79
12	Behavioural outcome	1·00	1·00	0·90	1·66
	The whole instrument	0·97	0·92	0·84	1·77

### Step 3 (content validity)

All opinions and corrective views of experts in the qualitative section of content validity were applied to the items. In the quantitative section, CVR and CVI showed that one item in the knowledge section, one item in the construct of previous relevant behaviour, two items in the construct of perceived benefits and one item in the construct of perceived barriers had CVR scores of <0·59. In addition, one item in the knowledge section and one item in the construct of perceived benefits had CVI scores of <0·70; therefore, these items were removed from the instrument. Moreover, one item in the construct of previous relevant behaviour and one item in the construct of behaviour-related emotions had CVI scores within the 0·70–0·79 range. Thus, these items were revised (Table 2). The mean CVR for all instrument items was 0·92. The highest mean CVR (1·00) pertained to the constructs of behavioural outcome and the commitment to the action plan, while the lowest (0·85) to the constructs of perceived self-efficiency and immediate preferences and demand (Table 3). The mean CVI for all instrument items was 0·97. The highest mean CVI (1·00) pertained to the constructs of motivational factors, behavioural outcome and commitment to the action plan and the lowest (0·93) to the constructs of previous relevant behaviour and perceived self-efficacy (Table 2). At the end of this step, 6·25 % of items dropout of the questionnaire.

**Table 3.** Demographic characteristics

	Study group		Iran population	
Variable		Mean	*N* (%)	Mean	%
Age		7·56±40·25		31·10±4·42	
Gender	Male		238(56·90)		50·66
	Female		180(43·10)		49·33
Residence	Urban		368(88·00)		74·04
	Rural		50(12·00)		25·95
Marital status	Single (never not married)		58(13·90)		30·35
	Married		353(84·40)		63·86
	Others^a^		7(1·70)		5·78
Education	Middle school		3(0·70)		25·70
	High school		2(0·50)		12·52
	High school diploma		50(12·00)		30·97
	College degree		363(86·80)		70·79
Employment status	Contractual		125(29·90)		13·52
	Semi-official		73(17·50)		13·14
	Official		173(41·40)		63·39
	Corporative recruitment		47(11·20)		5·93
Family size^b^	1–2		87(20·80)		
	3–4		285(68·20)		
	5–6		38(9·10)		
	>6		8(1·90)		
Household^c^ monthly revenue	<100		9(2·20)		
	100–200		134(32·10)		
	>200–300		141(33·70)		
	>300–400		106(25·40)		
	>400		28(6·70)		

a For example, divorced, widow, living apart from spouse.

b People.

c US$.

### Step 4 (internal consistency)

Cronbach's alpha coefficient was applied to measure reliability. The results indicated that the reliability coefficient of the knowledge section was <0·70, which was unacceptable. Accordingly, an item efficient in reducing the reliability coefficient was removed, and Cronbach's alpha coefficient reached an acceptable level. Cronbach's alpha coefficient was acceptable for other constructs. The highest Cronbach's alpha coefficient (0·92) pertained to the constructs of behaviour-related emotions and perceived barriers, while the lowest (0·71) was related to the construct of motivational factors. Cronbach's alpha coefficient of the whole instrument was 0·84 (Table 2).

### Step 5 (construct validity)

Analysis of Exploratory factor was performed on collected data from 418 questionnaires of the target group (government employees). The data of seven questionnaires were excluded due to incompleteness. The mean age of participants was 40·25 ± 7·56 years within the 26–60 range; other demographic characteristics are listed in Table 3.

**Fig. 2.**
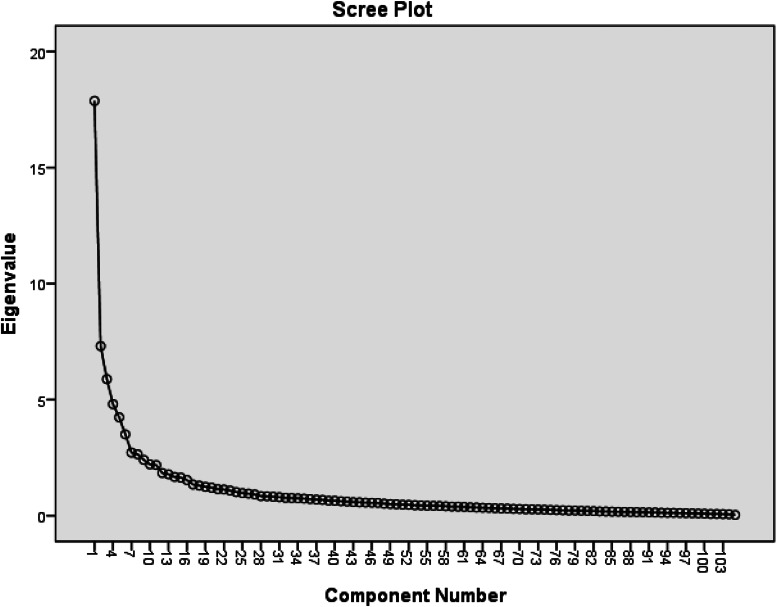
Pebble chart for image of the eigen value in each of the extracted items.

A KMO index of 0·85 was considered for the sample size adequacy. It showed that the sample size was suitable for exploratory factor analysis. The Bartlett's test of sphericity was significant (*P* < 0·001, *χ*^2^ = 87 985/874·87, df = 6903), showing a sufficient correlation between variables. According to the results of PCA with varimax rotation and minimum eigen value (Minimum one), 104 items were identified in the form of 15 factors extracted through exploratory factor analysis (Fig. 2). The perceived self-efficacy factor, accounting for 6·65 % of the model variance, was the strongest predictor and motivational factors (2), accounting for 2·43 % of the model variance, was the weakest predictor in the model. The factor loads of the items ranged from 0·40 to 0·83, and the cumulative variance for the whole model was 61·17 %. After factor analysis, reliability was calculated through the Cronbach's alpha coefficient for each factor. The factors behaviour-related emotions and perceived benefits had the highest Cronbach's reliability coefficient (0·96), while the factor behavioural outcome had the lowest Cronbach's reliability coefficient (0·72). The reliability coefficient for all factors was 0·96 (Table 4). At the end of this step, 11·86 % of items dropout of the questionnaire.

**Table 4.** Factor loads extracted from exploratory factor analysis with varimax rotation


	Factor 1			
	Perceived self-efficiency	CVR	CVI	Impact score
Items	Factor load			

I can plan to eat more fruit next week.	0·77	0·81	1·00	1·59
I can plan to eat more vegetables next week.	0·74	1·00	1·00	1·61
Despite my hectic schedule, I can eat five units of fruits and vegetables (two units of fruits and three units of vegetables) daily.	0·74	0·63	1·00	1·51
I can economically plan to buy more fruits and vegetables.	0·71	0·81	0·96	1·63
I can use fruits or vegetables even when I am upset or angry.	0·69	1·00	0·81	1·90
I can use fruit as a snack in the office.	0·66	0·81	0·96	2·16
I can use fruits and vegetables per day even during the cold season.	0·66	0·63	0·93	2·01
I can eat at least three units of vegetables per day (such as vegetables, cucumbers, tomatoes and carrots)	0·65	0·93	1·00	1·76
I can use vegetables (such as cucumbers, tomatoes and carrots) as a snack in the office.	0·61	0·81	0·96	1·61
I can eat at least two units of fruits a day.	0·58	1·00	0·87	2·16
I can eat fruits or vegetables (such as cucumbers, tomatoes and carrots) when I am outdoors (e.g. for shopping, leisure, travel).	0·57	0·81	0·93	1·63
Factor load range	0·57−0·77			
Eigen value	17·87			
Variance	6·65			
Cumulative variance	6·65			
Cronbach's alpha coefficient	0·92			

Items	Factor 2			
	Perceived barriers	CVR	CVI	Impact score
	Factor load			

Difficult access to greengrocers prevents me from consuming the required amount of vegetables.	0·78	1·00	1·00	1·61
Low quality and freshness of fruits in greengrocers prevents me from consuming the required amount of fruits.	0·77	0·81	1·00	1·65
Difficult access to greengrocers prevents me from consuming the required amount of fruits.	0·75	1·00	1·00	1·58
Low quality and freshness of vegetables in greengrocers prevents me from consuming the required amount of vegetables.	0·74	0·81	1·00	1·51
Fear of parasitic diseases due to the F&V intake prevents me from consuming the required amount of fruits and vegetables.	0·73	0·81	.93	1·86
Fear of pesticides used in the growth and maintenance of fruits and vegetables prevents me from consuming the required amount of fruits and vegetables.	0·68	1·00	1·00	1·91
Lack of enough time to buy fruits and vegetables prevents me from consuming the required amount of fruits and vegetables.	0·64	1·00	1·00	1·59
Lack of sufficient space to store large quantities of fruits and vegetables for several meals at home prevents me from consuming the required amount of fruits and vegetables.	0·62	0·81	1·00	1·54
Lack of enough information on preparation of fruits and vegetables (washing, disinfecting, peeling, chopping, preparing desserts and foods from them) prevents me from consuming the required amount of fruits and vegetables.	0·60	0·81	0·93	1·58
No F&V intake by my family prevents me from consuming the required amount of fruits and vegetables.	0·60	1·00	0·96	1·58
The time-consuming preparation of fruits and vegetables (washing, disinfecting, peeling, chopping, preparing desserts and foods from them) prevents me from consuming the required amount of fruits and vegetables.	0·59	1·00	1·00	1·55
No F&V intake by colleagues in the office prevents me from consuming the required amount of fruits and vegetables.	0·57	1·00	0·96	1·58
Lack of sufficient space to store F&V intake in the office prevents me from consuming the required amount of fruits and vegetables.	0·50	0·81	1·00	1·53
The price of fresh vegetables prevents me from consuming the required amount of vegetables.	0·47	1·00	1·00	2·36
The price of fresh fruits prevents me from consuming the required amount of fruits.	0·43	1·00	1·00	2·35
Factor load range	0·43−0·78			
Eigen value	7·30			
Variance	6·36			
Cumulative variance	13·01			
Cronbach's alpha coefficient	0·86			
	Factor 3			
Items	Situational influences (1) Factor load	CVR	CVI	Impact score

How much does each of the following places or social events affect your desire to eat vegetables? Parties.	0·79	1·00	1·00	1·58
How much does each of the following places or social events affect your desire to eat fruits? Parties.	0·78	1·00	1·00	1·58
How much does each of the following places or social events affect your desire to eat vegetables? Restaurant.	0·76	0·81	0·96	1·56
How much does each of the following places or social events affect your desire to eat fruits? Restaurant.	0·75	0·81	0·96	1·56
How much does each of the following places or social events affect your desire to eat vegetables? Holidays or occasions.	0·73	1·00	1·00	1·51
How much does each of the following places or social events affect your desire to eat fruits? Holidays or occasions.	0·69	1·00	1·00	1·63
How much does each of the following places or social events affect your desire to eat vegetables? Work place.	0·68	1·00	1·00	0·73
How much does each of the following places or social events affect your desire to eat fruits? Work place.	0·68	1·00	1·00	1·65
How much does each of the following places or social events affect your desire to eat vegetables? Friends’ house.	0·66	1·00	1·00	1·63
How much does each of the following places or social events affect your desire to eat fruits? Friends’ house.	0·63	1·00	1·00	1·58
Factor load range	0·63−0·79			
Eigen value	5·88			
Variance	5·73			
Cumulative variance	18·75			
Cronbach's alpha coefficient	0·93			

Items	Factor 4			
	Interpersonal influences (1)	CVR	CVI	Impact score
	Factor load			

How important is the opinion of your friends for you to consume more fruits and vegetables, or how much do you try to act according to their opinion?	0·83	1·00	1·00	1·51
How important are the opinions of your family and acquaintances for you to consume more fruits and vegetables, or how much do you try to act according to their opinion?	0·81	1·00	1·00	1·51
How important are the opinions of the office colleagues important for you to consume more fruits and vegetables, or how much do you try to act according to their opinion?	0·79	1·00	1·00	1·54
How important are the opinions of the treating doctor and healthcare providers for you to consume more fruits and vegetables, or how much do you try to act according to their opinion?	0·77	1·00	1·00	1·83
How important are the opinions of the health centre employees for you to consume more fruits and vegetables, or how much do you try to act according to their opinion?	0·77	1·00	0·96	1·98
How important are the opinions of people with valuable ideas (such as office head, athletes and celebrities) for you to consume more fruits and vegetables, or how much do you try to act according to their opinion?	0·75	0·81	1·00	1·51
How important is the opinion of family for you to consume more fruits and vegetables, or how much do you try to act according to their opinions?	0·64	1·00	1·00	1·71
Factor load range	0·64−0·83			
Eigen value	4·80			
Variance	4·54			
Cumulative variance	23·29			
Cronbach's alpha coefficient	0·92			
Item	Factor 5			
	Incentive factors1	CVR	CVI	Impact score
	Factor load			

How important is good taste to motivate you more to eat fruits and vegetables?	0·78	0·81	1·00	2·48
How important is freshness to motivate you more to eat fruits and vegetables?	0·77	1·00	1·00	2·50
How important is healthiness to motivate you more to eat fruits and vegetables?	0·73	1·00	1·00	2·50
How important is easy access to motivate you more to eat fruits and vegetables?	0·64	1·00	1·00	2·38
How important is proper price to motivate you more to eat fruits and vegetables?	0·60	1·00	1·00	2·50
How important is family consumption to motivate you more to eat fruits and vegetables?	0·58	1·00	1·00	1·93
How important are appearance and packaging to motivate you more to eat fruits and vegetables?	0·56	0·81	1·00	1·73
Factor load range	0·56−0·78			
Eigen value	4·24			
Variance	4·20			
Cumulative variance	27·49			
Cronbach's alpha coefficient	0·86			

Items	Factor 6			
	Interpersonal influences (2)	CVR	CVI	Impact score
	Factor load			

Do your friends expect or encourage you to eat fruits and vegetables to maintain and improve your health?	0·76	1·00	1·00	1·58
Do your family and acquaintances expect or encourage you to eat fruits and vegetables to maintain and improve your health?	0·74	1·00	1·00	1·53
Do your office colleagues expect or encourage you to eat fruits and vegetables to maintain and improve your health?	0·73	1·00	1·00	1·51
Do people with valuable opinion (such as office head, athletes, celebrities) expect or encourage you to eat fruits and vegetables to maintain and improve your health?	0·71	1·00	0·96	1·54
Do health centre employees expect or encourage you to eat fruits and vegetables to maintain and improve your health?	0·69	1·00	1·00	1·59
Do the treating doctor and healthcare providers expect or encourage you to eat fruits and vegetables to maintain and improve your health?	0·69	1·00	1·00	1·74
Do your family expect or encourage you to eat fruits and vegetables to maintain and improve your health?	0·47	1·00	1·00	1·66
Factor load range	0·47−0·76			
Eigen value	3·50			
Variance	4·10			
Cumulative variance	31·60			
Cronbach's alpha coefficient	0·92			

Items	Factor 7			
	Behaviour-related emotions	CVR	CVI	Impact score
	Factor load			

I feel cheerful and happy with eating vegetables.	0·74	1·00	0·93	1·66
I enjoy eating vegetables because of the variety it provides to the diet.	0·72	0·81	0·75	1·61
I feel cheerful and happy with eating fruits.	0·71	1·00	0·93	1·66
I like the taste of vegetables.	0·67	0·81	0·93	1·89
I enjoy eating fruits because of the variety it provides to the diet.	0·64	0·81	0·75	1·61
I like the taste of fruits.	0·63	1·00	1·00	1·61
F&V intake is valuable to me because they meet the essential needs of the body.	0·46	0·81	0·84	1·73
Factor load range	0·46−0·74			
Eigen value	2·71			
Variance	4·10			
Cumulative variance	35·71			
Cronbach's alpha coefficient	0·96			
Items	Factor 8			
	Perceived benefits	CVR	CVI	Impact score
	Factor load			

Daily F&V intake prevents premature aging of my skin by refreshing the face and skin.	0·73	0·81	1·00	1·93
Daily F&V intake saves on my medical expenses in the long run.	0·72	1·00	1·00	1·88
Daily F&V intake prevents me against chronic diseases such as cardiovascular disease, cancer, and diabetes.	0·67	1·00	1·00	1·99
Daily F&V intake balances my body weight.	0·67	1·00	0·96	2·03
Daily F&V intake eliminates the use of worthless snacks during the day by me.	0·64	0·81	1·00	1·55
Daily F&V intake enables me to better perform my activities.	0·63	1·00	0·96	1·55
Daily F&V intake provides my body with minerals and vitamins.	0·44	1·00	1·00	`1·56
Factor load range	0·44−0·73			
Eigen value	2·64			
Variance	4·01			
Cumulative variance	39·73			
Cronbach's alpha coefficient	0·96			

Items	Factor 9			
	Previous relevant behaviour	CVR	CVI	Impact score
	Factor load			

I eat fruits and vegetables at certain meals throughout the day.	0·72	0·81	0·87	1·81
I have eaten fruits or vegetables (such as cucumbers, tomatoes, carrots) while I was busy during the day (watching TV, reading, and working).	0·71	1·00	0·93	1·75
I have eaten fruits or vegetables (such as cucumbers, tomatoes, carrots) as snacks throughout the day.	0·70	1·00	1·00	1·88
I have eaten the recommended amount of fruits and vegetables (three to five units daily) in several meals throughout the day.	0·70	0·63	0·78	1·96
I have kept fruits or vegetables for consumption throughout the day.	0·70	0·81	0·91	1·73
In addition to meals, I have eaten vegetables (raw or in the form of salads).	0·62	1·00	0·90	1·86
I have eaten natural juice throughout the day.	0·44	1·00	0·96	1·66
Instead of eating sweets and biscuits, I have eaten fruits or vegetables (such as cucumbers, tomatoes and carrots) as snacks in the office.	0·40	1·00	1·00	1·56
Factor load range	0·40−0·72			
Eigen value	2·40			
Variance	4·01			
Cumulative variance	43·74			
Cronbach's alpha coefficient	0·87			

Items	Factor 10			
	Commitment to the action plan	CVR	CVI	Impact score
	Factor load			

Do you have a regular plan to consume the recommended amount of fruits throughout the day?	0·83	1·00	1·00	1·93
Do you have a regular plan to consume the recommended amount of vegetables throughout the day?	0·83	1·00	1·00	1·93
If yes, to what extent are you committed to your plan (fruits)?	0·83	1·00	1·00	1·63
If yes, to what extent are you committed to your plan (vegetables)?	0·81	1·00	1·00	1·63
Factor load range	0·81−0·83			
Eigen value	2·21			
Variance	3·40			
Cumulative variance	47·14			
Cronbach's alpha coefficient	0·89			
Items	Factor 11			
	Immediate preferences and demand	CVR	CVI	Impact score
	Factor load			

If any of the following are available to you at the same time and you are free to choose one of them, which one would you prefer to consume? Fruits or junk food (e.g. chips, puffs, fruit leather … ).	0·69	0·81	1·00	2·03
If any of the following are available to you at the same time and you are free to choose one of them, which one would you prefer to consume? Vegetables (e.g. cucumber, tomato, lettuce … ) or junk food (e.g. chips, puffs, fruit leather … ).	0·68	0·81	0·90	1·85
If any of the following are available to you at the same time and you are free to choose one of them, which one would you prefer to consume? Vegetables (e.g. cucumber, tomato, lettuce) or drinks (e.g. soft drinks and industrial juices … ).	0·66	0·81	0·93	1·54
If any of the following are available to you at the same time and you are free to choose one of them, which one would you prefer to consume? Fruits or drinks (e.g. soft drinks and industrial juices … ).	0·63	0·81	1·00	1·85
If any of the following are available to you at the same time and you are free to choose one of them, which one would you prefer to consume? Vegetables (e.g. cucumber, tomato, lettuce … ) or sweets (for example cakes, biscuits … ).	0·60	0·81	1·00	1·63
If any of the following are available to you at the same time and you are free to choose one of them, which one would you prefer to consume? Fruits or sweets (e.g. cakes, biscuits … ).	0·44	1·00	1·00	2·03
Factor load range	0·44−0·69			
Eigen value	2·18			
Variance	3·10			
Cumulative variance	50·25			
Cronbach's alpha coefficient	0·82			

Items	Factor 12			
	Situational influences (2)	CVR	CVI	Impact score
	Factor load			

How much does watching or listening to a TV or radio programme about the benefits of eating fruits or vegetables affect your desire to consume fruits and vegetables?	0·80	1·00	1·00	1·83
How much does studying the benefits of eating fruits and vegetables affect your desire to consume fruits and vegetables?	0·73	1·00	1·00	1·54
How much does attending training classes on the benefits of eating fruit or vegetables in the office or other places affect your desire to consume fruits and vegetables?	0·71	0·81	1·00	1·51
How much does daily F&V intake in the recommended amount (at least two units of fruits and three units of vegetables) by family, relatives, friends, or colleagues affect your desire to consume fruits and vegetables?	0·59	0·81	1·00	1·59
Factor load range	0·58−0·80			
Eigen value	1·83			
Variance	3·05			
Cumulative variance	53·30			
Cronbach's alpha coefficient	0·94			

	Factor 13			
Items	Knowledge	CVR	CVI	Impact score

Factor load
Which of the following food groups have a lot of fibre?	0·68	1·00	0·96	2·09
Which of the following is one of the benefits of fibre in food?	0·63	0·81	0·93	1·91
Which of the following is the best source of vitamin C in the body?	0·62	0·81	0·96	2·16
Which statement is true about F&V intake?	0·55	0·81	1·00	1·96
Consumption of which of the following food groups can rejuvenate the skin and hair and prevent premature aging and wrinkles?	0·48	0·81	1·00	1·81
Factor load range	0·48−0·68			
Eigen value	1·78			
Variance	2·71			
Cumulative variance	56·02			
Cronbach's alpha coefficient	0·78			
	Factor 14			
Items	Behavioural outcome	CVR	CVI	Impact score

Factor load
I eat fruits during working hours in the workplace.	0·68	1·00	1·00	1·63
I eat vegetables (such as cucumbers, tomatoes, carrots) during working hours in the workplace.	0·60	1·00	1·00	1·56
I plan to eat more vegetables in the future.	0·45	1·00	1·00	1·83
I plan to eat more fruits in the future.	0·43	1·00	1·00	1·85
Factor load range	0·43−0·68			
Eigen value	1·66			
Variance	2·71			
Cumulative variance	58·74			
Cronbach's alpha coefficient	0·72			

Items	Factor 15			
	Incentive factors 2	CVR	CVI	Impact score
	Factor load			

How important is eating fruits and vegetables by friends to motivate you more to consume fruits and vegetables?	0·61	1·00	1·00	1·56
How important is advertising through TV, movies, and other media to motivate you more to consume fruits and vegetables?	0·58	1·00	1·00	1·56
Factor load range	0·58−0·61			
Eigen value	1·53			
Variance	2·43			
Cumulative variance	61·17			
Cronbach's alpha coefficient	0·85			

### Step 6 (external validity)

There was no significant relationship between the demographic characteristics of the target group (government employees) and the general population of Iran (*P* < 0·001; Table 5).

**Table 5.** Investigating the relationship between the characteristics of the studied sample (government employees) and the general population of Iran

Variable	Test	*P*-value
Age	One-Sample *T*-Test	*P* < 0·001
Gender	One-Sample Binomial Test	*P* < 0·006
Residence	One-Sample Binomial Test	*P* < 0·001
Marital status	One-Sample Binomial Test	*P* < 0·001
Employment status	One-Sample Chi-Square Test	*P* < 0·001
Education	One-Sample Chi-Square Test	*P* < 0·001

## Discussion

One of the evaluated models in recognising behaviours and creating new behaviours is Pender's HPM^(48)^. According to this model, people commit to behaviours that the perceived benefits and self-efficacy of that behaviour are high and the perceived barriers are low^(49)^. Strategies related to interpersonal and situational influences are also two important factors in behaviour change in Pender's HPM^(50)^. Interpersonal influencing factors include recognising the behaviours, beliefs or attitudes of others^(51)^. Family, peers, authorities and health-care providers are important interpersonal resources that can increase or decrease commitment to action^(49)^. Interpersonal influences have indirect effect in addition to direct effect. Indirect effect is through social pressure or persuasion to commit to a plan of action^(52)^. Situational influence is an approach that not only assesses individual perceptions and cognition of each situation and context but also how to behave as a facilitator or barrier to behaviour and includes perceptions of existing options, wants and needs, and environmental characteristics^(53)^. Which can be effective through an environment saturated with stimuli to perform the behaviours, such as a logo that represents the salient feature of a health behaviours^(52)^. Therefore, people should relate to and recognise health behaviours and direct them to their interpersonal interactions in order to act more in the direction of behavioural intention and to adapt themselves more to the cognitive symptoms related to the behaviour in question^(54)^. In this way, F&V intake as a health behaviour as well is influenced by a series of psychological, social and structural factors leading to certain beliefs and choices^(40)^. Considering people make food choices about 220 times a day^(55)^, targeting individual, interpersonal and environmental factors to increase fruit and vegetable intake should be a priority for public health interventions^(56)^. On the other hand, the value of educational programmes also depends on their effectiveness based on the appropriate utilisation of theories and models^(48)^. In this regard, the present study aimed to determine the validity and reliability of an instrument to assess the perceived F&V intake behaviour based on Pender's HPM. The initial instrument was developed with 112 items, which eventually increased to 118 items (before step 5: *Construct Validity*) because, according to experts and participants, some common items of the F&V intake behaviour with a higher importance for accurate response were divided into two separate items. The final instrument had acceptable content validity (CVR and CVI) and reliability scores. The mean impact score of all items was acceptable except for four items that were unable to gain the minimum acceptable mean impact score. This can be attributed to using qualitative methods in formal validity and applying the results on items before performing a quantitative step, something which could minimise difficulty, inconsistency or ambiguity in words and phrases of each item. This indicates that effective and timely determination of face validity and content validity, both qualitatively and quantitatively, can affect formulating and reviewing of the items and can hence have a valuable effect on the instrument design. The highest mean impact score pertained to the construct of motivational factors, which was added as a new construct to Pender's HPM. According to this construct, there are certain factors in the form of internal or external stimuli that can strongly motivating person to develop the desired health behaviour despite the existing barriers. Therefore, the construct of motivational factors had a special importance from the perspective of the study target group. CVR, CVI and reliability results indicated an optimal validity and reliability of the research instrument. Tajfard *et al.* conducted a similar study on nutritional behaviour of women based on Pender's HPM^(57)^. The comparison of the results of that study with those of this one showed that the present study had a higher mean CVR in the constructs of previous relevant behaviour, perceived self-efficacy, interpersonal influences and behavioural outcome, a lower mean CVR in the construct of situational influences, and an equal mean CVR in the construct of commitment to the action plan. In addition, the present study had a higher mean CVI score in the constructs of perceived barriers, perceived benefits, interpersonal influences, situational influences and behavioural outcome, a lower mean CVI score in the constructs of perceived self-efficacy and behaviour-related emotions, and an equal mean CVI score in the construct of previous relevant behaviour. Regarding the instrument reliability, the present study had a higher Cronbach's alpha coefficient in the constructs of situational influences and behavioural outcome, a lower Cronbach's alpha coefficient in the constructs of previous relevant behaviour, perceived self-efficacy and perceived benefits, and an equal Cronbach's alpha coefficient in the construct of behaviour-related emotions. Eating habits are of special importance in the F&V intake^(58)^. Gholami *et al.* also showed that eating habits had the greatest impact on F&V intake^(34)^. In the present study, the reliability coefficient of this construct was 0·85, which was optimal; however, it was lower than that of the Gholami (0·90) and Kasten (0·95) studies^(19,28)^. Other models and theories have also been applied to develop instruments regarding the predictors of F&V intake such as the theory of planned behaviour and the stages of change model. For instance, Babazadeh *et al.*^(59)^ and Narimani *et al.*^(60)^ developed their instruments based on the theory of planned behaviour and the stages of change model with reliability coefficients of 0·82 and 0·87, respectively. Therefore, considering the reliability coefficient in the present study (0·84), it can be stated that the reliability scores of instruments in these studies developed through three different theories or models were approximately equal. In a study by Jung *et al.* on F&V intake, the constructs of the instrument developed based on the theory of planned behaviour had a Cronbach's alpha coefficient of 80–94^(1)^. To determine the construct validity, the KMO index indicated the appropriateness of the sample size for exploratory factor analysis. In order to achieve better results in the present study, a cut-off point of 0·40 was used as a criterion for high correlation between items. The results demonstrated that the majority of items (88·13) had a high correlation and hence remained in the study, while 11·86 % of the items had a less correlation than the cut-off point and hence were removed. Extracted factors including: Factor 1 ‘perceived self-efficacy’ with 11 items, Factor 2 ‘perceived barriers’ with 15 items, Factor 3 ‘situational influences’ (1) with 10 items, Factor 4 ‘interpersonal influences’ (1) with 7 items, Factor 5 ‘motivational factors’ (1) with 7 items, Factor 6 ‘interpersonal influences’ (2) with 7 items, Factor 7 ‘behaviour-related emotions’ with 7 items, Factor 8 ‘perceived benefits’ with 7 items, Factor 9 ‘previous relevant behaviour’ with 8 items, Factor 10 ‘commitment to action plan’ with 4 items, Factor 11 ‘immediate preferences and demand’ with 6 items, Factor 12 ‘situational influences’ (2) with 4 items, Factor 13 ‘knowledge’ with 5 items, Factor 14 ‘behavioural outcome’ with 4 items and Factor 15 ‘motivational factors’ with 2 items. After exploratory factor analysis, some factors, including situational influences 1 and 2, interpersonal influences 1 and 2, and motivational factors 1 and 2, which had a common concept based on the main constructs of Pender's HPM, were combined. A total of twelve main factors were achieved. Optimal reliability of the factors, separately and in total, can be attributed to the formation and correct extraction of factors. At the same time, since these factors could contribute to 61·17 % of the total variance of the model, they have strong predictive effects on the model, e.g. referring to the extraction factor 4 and 6 as interpersonal influences 1 and 2, which could make up a total of 8·64 % of the total variance of the model, can indicate the predictive power of this construct in the present study in understanding consumer behaviour. F&V were in line with the results of a study by Delshad *et al.* In Tehran, Iran, which showed that government employees who had more interpersonal influence were more likely to engage in the health behaviour studied (stretching exercise)^(61)^. Accordingly, the results of Guadagnin *et al.* can be mentioned in relation to the importance of the construct validation for development of an accurate instrument. In the present study, 44 % of the main items about nutritional knowledge had low construct validity; therefore, they were not useful for evaluating the concepts of nutritional knowledge. This highlights the importance of construct validity^(62)^. Motivational factors were also analysed in the present study as a developmental construct in Pender's HPM. The results showed that this construct not only was accepted in face validity, content validity and reliability but also played a role in confirming the construct validity as two separate factors called motivational factors 1 and 2, contributing to 6·63 % of the total variance in the model. Therefore, it can be used along with other constructs of the model in future studies. Considering that one of the applications of exploratory factor analysis is to check the convergent validity of the items classified in each factor, so in the present study, the correlation coefficient of all extracted factor items was higher than the desired level of 0·4, which showed good internal consistency at the level of items of these factors^(63)^. The results of external validity in the present study showed that the demographic characteristics of the study group are not homogeneous with the general population of Iran, thus the results of the present study cannot be generalised to the population of Iran, various reasons can explain this. First, the researcher selected a special study group (government employees) from the general population of Ghaemshahr to study, which usually includes a specific age group (24–55 years) and a large percentage of these people have a university degree and Also, due to being employed in offices, most of these people live in urban areas. Second, the study sample was selected from a city located in the north of Iran. The purpose of Instrument Validation was to use in this group and finally in the population of the city staff in future studies, so it is somewhat predictable that the capability. It does not have generalisation to the general population and especially to a larger society such as the population of Iran, which included different age groups and, in turn, the level of education, marital status and various employment status that were related to age groups. according to the latest study. In Mazandaran province of Iran, that people did not consume enough F&V^(13)^, therefore effective and comprehensive interventions need to be planned and considered to improve that behaviour in that group. Because of the complexity of this behaviour, any intervention for changing F&V consumption should be planned and implemented based on understanding of determinants at both the environment and individual levels at first step. Hence, understanding the mediators that facilitate our target group in improving their F&V intake behaviour first step require a valid and reliable instrument. Thus, the present study aimed at design a reliable and valid instrument to measure determinants of F&V intake behaviour as the first step of a multi-phase intervention programme. Of course, after measuring the determinants of behaviour using designed instrument, F&V intake assessment using F&V frequency instrument before and after of effective interventions will be measured. In this time, we do not want to assess any relation between scores in the instrument and intakes of F&V.

Participation of a large number of government employees and diversity of the designated offices were considered the strengths of the present study. The offices were located in different geographical locations of the city, paid different salaries and differed in terms of extent and performance; therefore, their employees were characterised by a wide range of working conditions. The large number of the instrument items was one of the research limitations. This could reduce the accuracy of responses. In addition, since the employees were only available at working hours, they hardly accepted to participate in the study or completed the questionnaire on time.

## Conclusion

In order to implement effective interventions for promoting health behaviour, it is necessary to identify the effective factors. This requires the use of appropriate, complete and accurate tools. As a result, time, accuracy and scientific principles for development and standardisation of instrument can guarantee the effectiveness of researchers’ attempts at promoting and enhancing the health of society. The research instrument can be used as a suitable instrument in future studies because it was developed in cooperation with health education experts and the target group (governmental employees) observing the scientific principles and gained desirable formal validity, content validity and reliability, and an acceptable construct validity through exploratory factor analysis.

## Supplementary material

For supplementary material accompanying this paper visit https://doi.org/10.1017/jns.2021.90. click here to view supplementary material
