# Supplementary material for: Validation of an instrument for perceived factors affecting fruit and vegetable intake based on Pender's health promotion model
Source: J Nutr Sci. 2022 Feb 9;11:e7. doi: 10.1017/jns.2021.90 (PMC8889231; doi:10.1017/jns.2021.90)
Supplement: Supplementary file 1 [file S2048679021000902sup001.docx]

**Questionnaire**

**Knowledge**

**Please select the correct option**

1. Which of the following food groups have a lot of fibre?

a. Milk and dairy □

b. Vegetables and fruits□

c. Meat □

d. Fat and sweets□

2. Which of the following food groups have a lot of fibre?

a. Weight loss□

b. Prevention of diabetes □

c. Prevention of constipation□

d. All cases□

3. Which of the following is the best source of vitamin C in the body?

a. Bread and cereals group □

b. Meat and legumes group□

c. Fruits and vegetables group □

d. Milk and dairy group□

4. Which statement is true about F&V intake?

1. Consumption of vegetables and fruits play an important role in the prevention and treatment of various cancers and cardiovascular diseases. □
2. Consumption of vegetables and fruits increases blood lipids. □
3. Consumption of vegetables and fruits provides energy and protein needed by the body. □

d. Consumption of fruits and vegetables causes anemia.□

5. Consumption of which of the following food groups can rejuvenate the skin and hair and prevent premature aging and wrinkles?

1. Milk and dairy group□
2. Vegetables and fruits group□
3. Meat and legumes group □
4. Bread and cereals group □

**Previous relevant behaviour**

| **Please indicate to what extent have you performed any of the following behaviors over the past month?** | **Never** | **Sometimes** | **Often** | **Always** |
| --- | --- | --- | --- | --- |
| 1. I eat fruits and vegetables at certain meals throughout the day. |  |  |  |  |
| 1. I have eaten fruits or vegetables (such as cucumbers, tomatoes, carrots) while I was busy during the day (watching TV, reading, and working). |  |  |  |  |
| 1. I have eaten fruits or vegetables (such as cucumbers, tomatoes, carrots) as snacks throughout the day. |  |  |  |  |
| 1. I have eaten the recommended amount of fruits and vegetables (3 to 5 units daily) in several meals throughout the day. |  |  |  |  |
| 1. I have kept fruits or vegetables for consumption throughout the day. |  |  |  |  |
| 1. In addition to meals, I have eaten vegetables (raw or in the form of salads). |  |  |  |  |
| 1. I have eaten natural juice throughout the day. |  |  |  |  |
| 1. Instead of eating sweets and biscuits, I have eaten fruits or vegetables (such as cucumbers, tomatoes, carrots) as snacks in the office. |  |  |  |  |

**Perceived self-efficiency**

| **Please, regardless of whether you really do these things or not, and considering all your limitations and possibilities, how much do you think you are able to do the following activities?** | **Strongly Disagree** | **Disagree** | **Neutral** | **Agree** | **Strongly Agree** |
| --- | --- | --- | --- | --- | --- |
| 1. I can plan to eat more fruit next week. |  |  |  |  |  |
| 1. I can plan to eat more vegetables next week. |  |  |  |  |  |
| 1. Despite my hectic schedule, I can eat 5 units of fruits and vegetables (two units of fruits and three units of vegetables) daily. |  |  |  |  |  |
| 1. I can economically plan to buy more fruits and vegetables. |  |  |  |  |  |
| 1. I can use fruits or vegetables even when I am upset or angry. |  |  |  |  |  |
| 1. I can use fruit as a snack in the office. |  |  |  |  |  |
| 1. I can use fruits and vegetables per day even during the cold season. |  |  |  |  |  |
| 1. I can eat at least 3 units of vegetables per day (such as vegetables, cucumbers, tomatoes, carrots) |  |  |  |  |  |
| 1. I can use vegetables (such as cucumbers, tomatoes, carrots) as a snack in the office. |  |  |  |  |  |
| 1. I can eat at least 2 units of fruits a day. |  |  |  |  |  |
| 1. I can eat fruits or vegetables (such as cucumbers, tomatoes, carrots) when I am outdoors (e.g. for shopping, leisure, travel). |  |  |  |  |  |

**Behavior-related emotions**

| **Please specify how you feel, before, during or after eating fruit or vegetables**. | **Never** | **a little** | **To somewhat** | **High** | **Very high** |
| --- | --- | --- | --- | --- | --- |
| 1. I feel cheerful and happy with eating vegetables. |  |  |  |  |  |
| 1. I enjoy eating vegetables because of the variety it provides to the diet. |  |  |  |  |  |
| 1. I feel cheerful and happy with eating fruits. |  |  |  |  |  |
| 1. I like the taste of vegetables. |  |  |  |  |  |
| 1. I enjoy eating fruits because of the variety it provides to the diet. |  |  |  |  |  |
| 1. I like the taste of fruits. |  |  |  |  |  |
| 1. F&V intake is valuable to me because they meet the essential needs of the body. |  |  |  |  |  |

**Perceived benefits**

| **Please indicate your agreement or disagreement with the following statements.** | **Strongly Disagree** | **Disagree** | **Neutral** | **Agree** | **Strongly Agree** |
| --- | --- | --- | --- | --- | --- |
| 1. Daily F&V intake prevents premature aging of my skin by refreshing the face and skin. |  |  |  |  |  |
| 1. Daily F&V intake saves on my medical expenses in the long run. |  |  |  |  |  |
| 1. Daily F&V intake prevents me against chronic diseases such as cardiovascular disease, cancer, and diabetes. |  |  |  |  |  |
| 1. Daily F&V intake balances my body weight. |  |  |  |  |  |
| 1. Daily F&V intake eliminates the use of worthless snacks during the day by me. |  |  |  |  |  |
| 1. Daily F&V intake enables me to better perform my activities. |  |  |  |  |  |
| 1. Daily F&V intake provides my body with minerals and vitamins. |  |  |  |  |  |

**Perceived barriers**

| **Please specify to what extent the following causes you not to consume the required amount of fruits and vegetables?** | **Never** | | **a little** | **to somewhat** | **High** | **Very high** |
| --- | --- | --- | --- | --- | --- | --- |
| 1. Difficult access to greengrocers prevents me from consuming the required amount of vegetables. |  | |  |  |  |  |
| 2. Low quality and freshness of fruits in greengrocersprevents me from consuming the required amount of fruits. |  | |  |  |  |  |
| 3. Difficult access to greengrocers prevents me from consuming the required amount of fruits. |  |  | |  |  |  |
| 4. Low quality and freshness of vegetables in greengrocers prevents me from consuming the required amount of vegetables. |  |  | |  |  |  |
| 5. Fear of parasitic diseases due to the F&V intake prevents me from consuming the required amount of fruits and vegetables. |  |  | |  |  |  |
| 6. Fear of pesticides used in the growth and maintenance of fruits and vegetables prevents me from consuming the required amount of fruits and vegetables. |  |  | |  |  |  |
| 7. Lack of enough time to buy fruits and vegetables prevents me from consuming the required amount of fruits and vegetables. |  |  | |  |  |  |
| 8. Lack of sufficient space to store large quantities of fruits and vegetables for several meals at home prevents me from consuming the required amount of fruits and vegetables. |  |  | |  |  |  |
| 9. Lack of enough information on preparation of fruits and vegetables (washing, disinfecting, peeling, chopping, preparing desserts and foods from them) prevents me from consuming the required amount of fruits and vegetables. |  |  | |  |  |  |
| 10. No F&V intake by my family prevents me from consuming the required amount of fruits and vegetables. |  |  | |  |  |  |
| 11. The time-consuming preparation of fruits and vegetables (washing, disinfecting, peeling, chopping, preparing desserts and foods from them) prevents me from consuming the required amount of fruits and vegetables. |  |  | |  |  |  |
| 12. No F&V intake by colleagues in the office prevents me from consuming the required amount of fruits and vegetables. |  |  | |  |  |  |
| 13. Lack of sufficient space to store F&V intake in the office prevents me from consuming the required amount of fruits and vegetables. |  |  | |  |  |  |
| 14. The price of fresh vegetables prevents me from consuming the required amount of vegetables. |  |  | |  |  |  |
| 15. The price of fresh fruits prevents me from consuming the required amount of fruits. |  |  | |  |  |  |

**Interpersonal influences**

| 1. **To consume more fruits and vegetables, how important is the opinion of the following people to you or do you try to act in accordance with their opinion?** | | **Always** | | **More often** | | **Often** | | **Very little** | | **Not at all** | |
| --- | --- | --- | --- | --- | --- | --- | --- | --- | --- | --- | --- |
| 1. Friends | |  | |  | |  | |  | |  | |
| 1. Your family and acquaintances | |  | |  | |  | |  | |  | |
| 1. Office colleagues | |  | |  | |  | |  | |  | |
| 1. The treating doctor and healthcare providers | |  | |  | |  | |  | |  | |
| 1. The health centre employees | |  | |  | |  | |  | |  | |
| 1. The people with valuable ideas (such as office head, athletes, celebrities) | |  | |  | |  | |  | |  | |
| 1. Family | |  | |  | |  | |  | |  | |
| **b. Do the following people expect you to eat fruits and vegetables to maintain and improve your health, or do they encourage you to eat them?** | | **Strongly Disagree** | | **Disagree** | | **Neutral** | | **Agree** | | **Strongly Agree** | |
| 1. Friends | |  | |  | |  | |  | |  | |
| 1. Your family and acquaintances | |  | |  | |  | |  | |  | |
| 1. Your office colleagues | |  | |  | |  | |  | |  | |
| 1. The people with valuable opinion (such as office head, athletes, celebrities) | |  | |  | |  | |  | |  | |
| 1. The health centre employees | |  | |  | |  | |  | |  | |
| 1. The treating doctor and healthcare providers | |  | |  | |  | |  | |  | |
| 1. Your family | |  | |  | |  | |  | |  | |

**Situational influences**

| 1. **How much does each of the following places or social events affect your desire to eat fruit and vegetables?** | | **Always** | | **More often** | | **Often** | | **Very little** | | **Not at all** | |
| --- | --- | --- | --- | --- | --- | --- | --- | --- | --- | --- | --- |
| 1. How much does each of the following places or social events affect your desire to eat vegetables? Parties. | |  | |  | |  | |  | |  | |
| 1. How much does each of the following places or social events affect your desire to eat fruits? Parties. | |  | |  | |  | |  | |  | |
| 1. How much does each of the following places or social events affect your desire to eat vegetables? Restaurant. | |  | |  | |  | |  | |  | |
| 1. How much does each of the following places or social events affect your desire to eat fruits? Restaurant. | |  | |  | |  | |  | |  | |
| 1. How much does each of the following places or social events affect your desire to eat vegetables? Holidays or occasions. | |  | |  | |  | |  | |  | |
| 1. How much does each of the following places or social events affect your desire to eat fruits? Holidays or occasions. | |  | |  | |  | |  | |  | |
| 7. How much does each of the following places or social events affect your desire to eat vegetables? Work place. | |  | |  | |  | |  | |  | |
| 8. How much does each of the following places or social events affect your desire to eat fruits? Work place. | |  | |  | |  | |  | |  | |
| 9. How much does each of the following places or social events affect your desire to eat vegetables? Friends’ house. | |  | |  | |  | |  | |  | |
| 10. How much does each of the following places or social events affect your desire to eat fruits? Friends’ house. | |  | |  | |  | |  | |  | |
| 1. **Please specify to what extent affecting your desire to consume fruits and vegetables in any of the following situations and conditions?** | | **Always** | | **More often** | | **Often** | | **Very little** | | **Not at all** | |
| 1. How much does watching or listening to a TV or radio program about the benefits of eating fruits or vegetables affect your desire to consume fruits and vegetables? | |  | |  | |  | |  | |  | |
| 1. How much does studying the benefits of eating fruits and vegetables affect your desire to consume fruits and vegetables? | |  | |  | |  | |  | |  | |
| 1. How much does attending training classes on the benefits of eating fruit or vegetables in the office or other places affect your desire to consume fruits and vegetables? | |  | |  | |  | |  | |  | |
| 1. How much does daily F&V intake in the recommended amount (at least two units of fruits and three units of vegetables) by family, relatives, friends, or colleagues affect your desire to consume fruits and vegetables? | |  | |  | |  | |  | |  | |

**Incentive factors**

| **How important is each of the following to motivate you more to eat fruits and vegetables?** | | **It does not important at all** | | **It important very little** | | **Neutral** | | **It important** | | **It important very much** | |
| --- | --- | --- | --- | --- | --- | --- | --- | --- | --- | --- | --- |
| 1. Good taste | |  | |  | |  | |  | |  | |
| 1. Freshness | |  | |  | |  | |  | |  | |
| 1. Healthiness | |  | |  | |  | |  | |  | |
| 1. Easy access | |  | |  | |  | |  | |  | |
| 1. Proper price | |  | |  | |  | |  | |  | |
| 1. Family consumption | |  | |  | |  | |  | |  | |
| 1. Appearance and packaging | |  | |  | |  | |  | |  | |
| 1. Eating fruits and vegetables by friends | |  | |  | |  | |  | |  | |
| 1. Advertising through TV, movies, and other media | |  | |  | |  | |  | |  | |

**Commitment to the action plan**

| **Please indicate how committed you are to eating the recommended amount of fruits and vegetables throughout the day** | **High** | **to somewhat** | **Not at all** |
| --- | --- | --- | --- |
| 1. Do you have a regular plan to consume the recommended amount of fruits throughout the day? |  |  |  |
| 1. Do you have a regular plan to consume the recommended amount of vegetables throughout the day? |  |  |  |
| 1. If yes, to what extent are you committed to your plan (fruits)? |  |  |  |
| 1. If yes, to what extent are you committed to your plan (vegetables)? |  |  |  |

**Immediate preferences and demand**

| **If any of the following are available to you at the same time and you are free to choose one of them, which one would you prefer to consume?** | | 1 | 2 |
| --- | --- | --- | --- |
| 1. Fruits 2. junk food (e.g., chips, puffs, fruit leather…). |  |  |  |
| 1. Vegetables (e.g., cucumber, tomato, lettuce,) 2. Junk food (e.g., chips, puffs, fruit leather,..). |  |  |  |
| 1. Vegetables (e.g., cucumber, tomato, lettuce) 2. Drinks (e.g., soft drinks and industrial juices,..). |  |  |  |
| 1. Fruits 2. Drinks (e.g., soft drinks and industrial juices…). |  |  |  |
| 1. Vegetables (e.g., cucumber, tomato, lettuce,) 2. Sweets (for example cakes, biscuits,..). |  |  |  |
| 1. Fruits 2. Sweets (e.g., cakes, biscuits,..). |  |  |  |

**Behavioral outcome**

| **Please specify how you do each of the following:** | **Always** | **More often** | **Often** | **Very little** | **Not at all** |
| --- | --- | --- | --- | --- | --- |
| I eat fruits during working hours in the workplace. |  |  |  |  |  |
| I eat vegetables (such as cucumbers, tomatoes, carrots) during working hours in the workplace. |  |  |  |  |  |
| I plan to eat more vegetables in the future. |  |  |  |  |  |
| I plan to eat more fruits in the future. |  |  |  |  |  |
